# Supplementary material for: Metabolic specialization drives reduced pathogenicity in Pseudomonas aeruginosa isolates from cystic fibrosis patients
Source: PLoS Biol. 2024 Aug 23;22(8):e3002781. doi: 10.1371/journal.pbio.3002781 (PMC11376529; doi:10.1371/journal.pbio.3002781)
Supplement: S1 Table — (DOCX) [file pbio.3002781.s013.docx]

**Supplementary Table 1. Strains and plasmids used in this study**

| **Strain** | **Genotype** | **Infection age (years)** | **Mutations difference** | **Source** |
| --- | --- | --- | --- | --- |
| DK03 Early | *P. aeruginosa 317* | *0* |  | Marvig et al., (2015) [39] |
| DK03 Late | *P. aeruginosa 309* | *3.8* | *45* | Marvig et al., (2015) [39] |
| DK12 Early | *P. aeruginosa 95* | *0* |  | Marvig et al., (2015) [39] |
| DK12 Late | *P. aeruginosa LRJ09* | *7.0* | *54* | Marvig et al., (2015) [39] |
| DK13 Early | *P. aeruginosa 122* | *0* |  | Marvig et al., (2015) [39] |
| DK13 Late | *P. aeruginosa LRJ06* | *7.1* | *45* | Marvig et al., (2015) [39] |
| DK15 Early | *P. aeruginosa 135* | *0* |  | Marvig et al., (2015) [39] |
| DK15 Late | *P. aeruginosa 141* | *3.7* | *51* | Marvig et al., (2015) [39] |
| DK17 Early | *P. aeruginosa li* | *0* |  | Marvig et al., (2015) [39] |
| DK17 Late | *P. aeruginosa B* | *2.1* | *43* | Marvig et al., (2015) [39] |
| DK36 Early | *P. aeruginosa 410* | *0* |  | Marvig et al., (2015) [39] |
| DK36 Late | *P. aeruginosa 404* | *2.7* | *402* | Marvig et al., (2015) [39] |
| DK41 Early | *P. aeruginosa 367* | *0* |  | Marvig et al., (2015) [39] |
| DK41 Late | *P. aeruginosa 359* | *1.4* | *40* | Marvig et al., (2015) [39] |
| DK55 Early | *P. aeruginosa LRJ27* | *0* |  | Bartell et al., (2019) [9] |
| DK55 Late | *P. aeruginosa LRJ30* | *1.8* |  | Bartell et al., (2019) [9] |
| **Strain** | **Genotype** | | | **Source** |
| PAO1 | *P. aeruginosa* PAO1 wild type | | | Lab stock |
| *aceE* | *P. aeruginosa* PAO1; *aceE* t551c | | | This work |
| *aceF* | *P. aeruginosa* PAO1; *aceF* +tccc 816-819 | | | This work |
| *ΔpscC* | *P. aeruginosa PAO1; ΔpscC* | | | This work |
| *aceE(rev)* | *P. aeruginosa* PAO1; *aceE* t551c; wild type *aceE* integration into *att*Tn7 site | | | This work |
| **Plasmid** | **Description** | | | **Source** |
| pCasPA | Vector carrying P_ara_-*cas9* and λ Red recombination system; Tet^R^ | | | [2] |
| pACRISPR | Template vector for homology flanks and sgRNA integration; Cab^R^ | | | [2] |
| pIP243 | Editing plasmid to introduce *aceF* +tccc 816-819; Cab^R^ | | | This work |
| pIP251 | Editing plasmid to introduce *aceE* t551c; Cab^R^ | | | This work |
| pIP266 | Editing plasmid to delete *pscC* gene | | | This work |
| pIP281 | Mini-Tn7 plasmid to integrate wild type *aceE* | | | This work |
