# Supplementary material for: Metabolic specialization drives reduced pathogenicity in Pseudomonas aeruginosa isolates from cystic fibrosis patients
Source: PLoS Biol. 2024 Aug 23;22(8):e3002781. doi: 10.1371/journal.pbio.3002781 (PMC11376529; doi:10.1371/journal.pbio.3002781)
Supplement: S2 Table — (DOCX) [file pbio.3002781.s014.docx]

**Supplementary Table 2. Primers used in this study**

| **Primer name** | **Sequence** | **Description** |
| --- | --- | --- |
| PNJ1377 | ACACAUTATACGAGCCGGATGATTAATTGTC | Reverse to amplify the backbone of pACRISPR |
| PNJ1378 | ACTTTTCAUACTCCCGCCATTCAGAAG | Forward to amplify the backbone of pACRISPR |
| PNJ1379 | AGACCAUGGGTATGGACAGATCTCA | Reverse to amplify sgRNA |
| PNJ1533 | ATGTGUGGGATGCGACCCTTGGGACCGCGTT TTAGAGCTAGAAATAGCAAGTTAAAATAA | Forward to amplify sgRNA, *aceF* +tccc |
| PNJ1534 | ATGGTCUAGAGCCAGCGTCCAGGACATCAAGG | Forward, *aceF* +tccc left flank |
| PNJ1535 | ACCGCUGGCTTTCACTTCGCTC | Reverse , *aceF* +tccc left flank |
| PNJ1536 | AGCGGUCCCTCCCAAGGGTCGCATCCTCAAGG | Forward, *aceF* +tccc right flank |
| PNJ1537 | ATGAAAAGUCTCGAGACCGGGACCAGCAGGCCGTC | Reverse , *aceF* +tccc right flank |
| PNJ1568 | ATGCCGAUATACTATGCCGATGATTAATTGTCAACAATTAATTAAAGG | Forward to amplify sgRNA *aceE* t551c |
| PNJ1569 | ATCGGCAUAGGTGGGGAACTGCCAGAAGTCGTTTTAGAGCTAGAAATAGCAAGTTAAAAT | Reverse to amplify sgRNA *aceE* t551c |
| PNJ1617 | ATGGTCUAGAAATGCCTCTGGCAACCTCTCTGCC | Forward, Δ*pscC* left flank |
| PNJ1618 | ATGCTAAUTTCAGGGACGCCACACCGGAGC | Reverse, Δ*pscC* left flank |
| PNJ1619 | ATTAGCAUGGCCTGGAAGATCCGCTTC | Forward, Δ*pscC* right flank |
| PNJ1620 | ATGAAAAGUCTCGAGTGCACATCCACCTCGCTCATGCC | Reverse, Δ*pscC* right flank |
| PNJ1629 | ATGTGUGGGTCACCCTGTTGCAGAGCCAGTTTTAGAGCTAGAAATAGCAAGTTAAAATAA | Forward to amplify sgRNA, Δ*pscC* |
| PNJ1634 | AGGTGCGGCAGATTCTCCTCTG | Sequence *pscC* deletion, fw |
| PNJ1635 | GAATTTCTGCAGGATGAAATCCACGC | Sequence *pscC* deletion, rv |
| PNJ1746 | ATCGAATUAGCTTCAAAAGCGCTCTGAAG | Forward to amplify the backbone of mini-Tn7 plasmid |
| PNJ1747 | AGGGGUGGTACCTCGCGAAGG | Reverse to amplify the backbone of mini-Tn7 plasmid |
| PNJ1748 | AATTCGAUCGAAAAGGCTCTACATCGGGCCTTATAGC | Forward, *aceE* |
| PNJ1752 | AGGTATUAGCAATCCAGCGGGTTGCGCTTC | Reverse, *aceE* |
| PNJ1750 | AATACCUGCGCCGCTCCCCAC | Forward, *aceF* terminator |
| PNJ1751 | ACCCCUGCAGTTTCGCCGAGCCGTCAG | Reverse, *aceF* terminator |
