# Supplementary material for: Metabolic specialization drives reduced pathogenicity in Pseudomonas aeruginosa isolates from cystic fibrosis patients
Source: PLoS Biol. 2024 Aug 23;22(8):e3002781. doi: 10.1371/journal.pbio.3002781 (PMC11376529; doi:10.1371/journal.pbio.3002781)
Supplement: S1 Text — (DOCX) [file pbio.3002781.s015.docx]

**Supplementary text**

*Metabolic preferences of late metabotypes*

Adapted metabotype 1 is associated with reduced assimilation of Lys, Gly, Ile, Leu, Phe, Thr, Tyr and Val, and secretion of pyruvate by strain DK55 late; metabotype 2 shows increased assimilation of glucose, Ala, Asp, Glu, Gly, Pro, Ser, Arg and His, and secretion of a high amount of pyruvate; and metabotype 3 shows increased assimilation of glucose and lactate, differential assimilation of Ala, Asp, Glu, His and Pro and secretion of pyruvate by strain DK36 late (Fig. 2c-d). Late DK13, DK36 and DK41 strains secrete a low amount of formate whereas all late isolates secrete acetate, albeit at different concentrations and with different growth-dependent profiles (Fig. 2d).

*Convergent proteomic changes in metabolic proteins*

Several enzymes belonging to the Entner-Doudoroff/Embden-Meyerhof-Parnas/Pentose Phosphate (EDEMP), and tricarboxylic acid (TCA) cycle pathways are up-regulated in the early and late strains relative to PAO1, indicating that enzymes involved in central carbon metabolism converge to a configuration that is optimal for exploiting the airway’s resources (Fig. 3e and S2). For example, our metabolomic data show that several late strains (belonging to metabotypes 2 and 3) exhibit increased glucose assimilation, which is in agreement with the increased expression of pathways involved in glucose assimilation (enzymes 3, 11, 12, 18) (Fig. 3e). Similarly, fumarate hydratase (enzyme 33) together with the enzymes involved in Trp, Phe and Tyr catabolism (enzymes 65 and 66) shows increased expression, likely to better accommodate the increased assimilation of amino acids catabolized through the TCA cycle (Fig. 3e). This result is corroborated by the changes in expression (both increased and decreased) in late *vs* early strains of several enzymes involved in amino acid, glucose, and lactate catabolism, and of their respective transporters (Fig. 3e). This proteome configuration supports the hypothesis that overflow of amino acids through central carbon metabolism might be the cause of increased pyruvate secretion in the late strains of DK12, DK36 and DK55 (Fig. 2d and 3e). Still, further analyses are needed to confirm whether changes in metabolic enzymes leading to metabolic specialization are convergent.
