# Supplementary material for: Metabolic specialization drives reduced pathogenicity in Pseudomonas aeruginosa isolates from cystic fibrosis patients
Source: PLoS Biol. 2024 Aug 23;22(8):e3002781. doi: 10.1371/journal.pbio.3002781 (PMC11376529; doi:10.1371/journal.pbio.3002781)
Supplement: Attachment — Submitted filename: Response to Reviewers.pdf [file pbio.3002781.s016.pdf]

Metabolic specialization drives reduced pathogenicity in *Pseudomonas aeruginosa* CF clinical isolates

We are grateful for the time and effort dedicated by the reviewers in critically evaluating our work. Their comprehensive feedback has been very useful for shaping the revised version of this manuscript. Our responses to all points raised by the reviewers are detailed below in blue.

Reviewer #1:

Pedersen et al. present a compelling manuscript examining if metabolic adaptation contributes to the persistence of P. aeruginosa (Pa) in chronic infections, like those in people with CF. The authors use a combination of metabolomics and proteomics to examine this concept using a well-characterized longitudinal collection of clinical isolates of Pa from pwCF. The manuscript is well written and the hypothesis is a new contribution to the field, especially as considered using the metabolomics approach with the clinical isolate collection, but there are a few comments for the authors consideration that if addressed, would strengthen the results presented.

Comments:

1. Overall, it is unclear the statistical methods used, if any, for a number of the observations. For example, for the metabotypes, which statistical tests allowed the authors to define these? For figure 3d, e, these are great visualizations of complex data. Were statistics performed on the highlighted phenotypes with differently regulated metabolites? If so, which are significant? Showing patterns in pathways is interesting, but statistics are needed to validate observations. This goes for most figures, please describe the statistical tests used for each figure, as they are likely quite different, given the many approaches used in the study. Without this information, it is very hard to rigorously review the study.

We acknowledge the comment from the reviewer, and we recognize that the statistical methods in our manuscript are not always easy to find and read. In material and methods under "Data analysis" (see line 670), we present the statistical methods altogether. To avoid confusion and make the specific analyses easier to find, we have included specific information on the statistical methods in each result section and figure legend.

Naïve and adapted metabotypes were designated by using PCA and iterative K-mean clustering first, followed by curation according to HCA. We have expanded the Data analysis section in the material and methods to include specific information on the metabotypes designation (see lines 686-690).

For figure 3d, to confirm that the difference in the number of differentially expressed proteins between clusters is significant, we performed two-sided paired t-test which showed a P value of 0.0047. We have included this information in the results, figure and figure legend (see lines 212-214).

As also suggested by reviewer 3, we moved figure 3e and its corresponding text to supplementary text 1 and supplementary figure S2. Moreover, we have specifically indicated that convergent proteomic changes at the pathway level require further analyses and confirmations.

We hope this clarifies the confusion and allows for easier recognition of the statistical method used.

2. Some terminology is confusing. The authors use the term "naïve" metabotypes to describe cluster A, yet this cluster has a combination of early and late isolates. The term "adapted" also is used for other metabotypes. These terms when applied without statistical tests shape the conclusions and need to be rigorous tested to be used. Especially as naïve vs adapted clusters are driving later experiments and greatly impacting the conclusions drawn from the study.

We agree with the reviewer that the use of the terms 'naïve' and 'adapted' both for describing metabotypes and proteomic changes creates confusion. We have, therefore, rephrased the results (see lines 203-210) and verified the used of this terminology in the entire manuscript. Moreover, for the HCA presented in figure 3 b and c, and all the other HCA in our manuscript, we used the statistical test of bootstrapping (10,000 replicates) with % of bootstrap values presented on the branches. Separation between cluster A and B is consistently shown in 99% of the replicates making us confident of the conclusions.

3. In figure 2b, what is driving the two clusters of late isolates? Can you deduce this from the variables driving PC2 in fig 2A?

The statistical analysis performed on the hierarchical clustering (bootstrap analysis for 10,000 replicates) indicates that this specific clustering reappears in only 18% of the replicates. This separation is therefore highly uncertain, meaning those strains could also be clustered together with the rest of the early strains as is the case in the PCA in Fig. 2a. For this reason, we are not sure of the specific drivers of the separation of DK17_L and DK41_L.

4. In figure 3, are there any patient characteristics unique to cluster B? Antibiotic regimen differences? Mucoidy of the isolates? Lung function?

Unfortunately, we do not have data on treatment or lung function. Therefore, we are unable to correlate patient data with specific clusters. None of the isolates are mucoid.

5. In figure 4b, what does the fold change look like for the early vs late isolates? This comparison seems very interesting to the study and would be nice to see, in addition to the PAO1 comparison shown.

This comparison is included in fig 4c for a set of virulence categories where statistically significant differences between early vs late and between clone types are present. We have specified this comparison in the results (see lines 244-248). Moreover, in fig 4a we show enrichment analysis for the comparison Late vs Early for the KEGG and GO terms. Finally, in Supplementary Data 2 we present all the fold changes for the Late vs Early comparison.

6. Lines 257-258, this statement seems like a testable hypothesis. This comment should be either be tested experimentally or moved to the discussion, if there is no data.

We agree with the reviewer that we do not have experimental data on the strains' immunogenicity. Therefore, we modified the sentence to underline the changes in virulence factors expression profiles rather than speculating on the strains' immunogenicity.

7. Also lines 356-358, this commentary is more appropriate for the discussion than in the results, as it doesn't set up data to be shown.

We agree with the reviewer's comment and therefore we have moved the commentary to the discussion. See lines 438-441.

8. In general, the authors are using the term immunogenicity, when they are just measuring IL-8 secretion. Inflammation is more descriptive of the results presented, since the authors are just focusing on IL-8.

Changed to inflammation.

9. The final paragraph in the results is interesting, but needs more experimental data to support the conclusions the authors are making. Does an aceE/F deletion mutant have altered T3SS activity? Changes in exotoxin gene expression? Mutations in both the PDHc pathway and T3SS in clinical isolates can be true and not related. Experimentation is needed to link these two in the strains discussed, if the authors want to present the conclusions in lines 373-376. Or move this commentary to the discussion section.

We agree with the reviewer's comment and therefore we have rephrased the conclusions of the paragraph and moved the commentary to the discussion. See lines 366-373 and 408-413.

10. What is the pyruvate concentration in CF sputum? Addition of this information would support the authors results for a role for pyruvate in establishing persistent Pa infections in CF.

Pyruvate has been identified in sputum samples during exacerbation events at an average concentration of 0.3 mM. We have included this information in the text. See lines 425-426.

11. For the comment in lines 450-452, were antibiotic resistance genes dysregulated in the aceE or aceF proteomics studies? If the metabolic phenotype precedes the development of antimicrobial resistance, you might expect antibiotic resistance gene expression to be altered in the aceE/F mutant backgrounds.

We did not find resistance genes with dysregulated expression. Moreover, the *aceE* and *aceF* strains do not show altered MICs relative to PAO1 wild type. We only found a slight increase in Tobramycin MIC which most likely depends on the reduced growth rate of the strains (McKenney et al., 1997 doi: 10.1093/jac/40.3.415; La Rosa et al., 2021 doi:10.1038/s41467-021-23451-y). Since in our collection of clinical strains (Bartell et al., 2019 doi:10.1038/s41467-019-08504-7) antibiotic resistance occurs only after years of treatment, we argue that adaptation to the host environment and specifically metabolic specialization might be more important at the beginning of the infection to counterbalance the host and its immune system.

Reviewer #2:

The manuscript by Pedersen et al tackles the role that metabolic adaptation plays in the cystic fibrosis context. The manuscript first uses a metabolomics approach to assess differences between early and late isolates from 8 patients. The metabolomic data suggests that late isolates often have similar metabolic profiles compared to their early counterparts. Proteomic data also supports similarity in late strains compared to early. Both metabolomic and proteomic analyses point to the secretion of pyruvate as a key factor associated with late strains. Analysis of the proteomic data suggests that late strains have predicted changes in virulence protein expression that agree with previous work. They also find mutations in genes that code for the pyruvate dehydrogenase complex (aceE and aceF). They then generate mutants in the PA01 background that harbor these mutations, which they then use for whole cell proteomics. The proteomic data suggest far reaching consequences for gene expression in these mutants, and they were defective for virulence as measured in an ALI model.

This is an interesting study that tries to tie together metabolic adaptation with other known adaptations, and is tantalizing in suggesting that metabolic changes likely precede other adaptations. The finding need to be supported with further clarifications, a clear exposition of limitations, and genetic complementation of the mutants. Overall the findings of this study, especially the metabolomics and proteomics could be presented much more clearly, and these sections could be shortened.

We thank the reviewer for the comment. We have addressed his/her concerns and clarified where requested (see below). We have expanded the discussion section to address further limitations of our study, and shortened the metabolomics and proteomics sections to introduce a supplementary text with a more extended and detailed presentation of the specific differences between the strains. Moreover, as requested, we have performed complementation assays of one of the mutant strains.

Major concerns:

1. It is clear that heterogeneous populations of PA can coexist for long periods of time in pwCF. Therefore, the selection of single isolates from early and late is a limitation unless the investigators can say definitively that the late strain was not present at the early timepoint. Likewise, the assumption that early isolates are not adapted to the CF airway should also be treated as a limitation.

We appreciate the reviewer's comment and suggestion on the heterogeneous populations present in pwCF. We previously compared the single isolates collection from certain patients to their corresponding metagenomic profile (Sommer et al., 2016 doi:10.1186/s12864-016-2873-1). Our data showed that single isolates collected from sputum samples are usually the most abundant representative of the population. This confirms that single isolate collections can be used to characterize the adaptive and evolutionary process of *P. aeruginosa* in pwCF. Indeed, while we do agree that pwCF are colonized by a heterogeneous population, we believe that single isolates well-represent the most abundant sub-population of the person. Moreover, the goal of our work is not to describe the metabolic changes occurring in the entire population, but rather to identify molecular mechanisms of metabolic adaptation which change the host-pathogen interactions. For this reason,

we prioritized to include different patients and different clone types instead of several isolates from the same patient. We have clarified this point in the result section (lines 150-154).

2. The selection of early and late strains is a bit unclear. In the results, it seems as if the late strains were specifically chosen based on decreased growth rates. This might bias the data and confuse "late" for "growth retardation".

As indicated in the first paragraph of the result section, growth rate reduction is a key phenotype associated with adaptive evolution in pwCF. Therefore, to identify new mechanisms of metabolic specialization occurring during within host evolution and to increase the likelihood of selecting interesting isolates, we chose late isolates which also had a reduced growth rate. This allowed us to put together a collection of early and late adapted strains where to analyse metabolic specialization. Still independently of the specific selected strains, we identified mechanisms of metabolic specialization occurring in late slow growing isolates which are used in different patients and infection scenarios to modulate the host response. We have rephrased most of the first result paragraph to clarify the strain selection approach (lines 126-154).

3. The authors do not address the phylogenetic relatedness of the strain and only refer to previous work in ref 36. However, it would be helpful to present that data here in order to understand how closely each of the strains is related to each other to understand whether the observed changes in metabolism are generalizable across PA. It would also help with interpretation of HCA and the clustering of metabolomic and proteomic profiles.

We acknowledge the request from the reviewer on the phylogenetic relationship between the strains. However, since the genome sequences of the clinical isolates have already been published and their relationship defined elsewhere (Marvig et al., 2014 doi:10.1038/ng.3148), we have not included this information here. Moreover, since each pair of early and late strains belongs to the same clone types and we examined pairs from several different pwCF, when performing phylogenetic analysis on the strain's mutations, they will always cluster based on their mutational signature which by definition of clone types is more than 10,000 (Marvig et al., 2014 doi:10.1038/ng.3148). This can be evidenced by a recent publication from our group where we performed phylogenetic analysis on isolates from different pwCF and different clone types (Espaillat et al., 2024 doi:10.1093/molbev/msae022, Fig. 1B).

4. The aceF and aceE mutants should be complemented especially since many of the tests are on phenotypes not directly related to the action of these two enzymes.

We acknowledge the comment from the reviewer, and we have performed complementation assays for the *aceE* mutant strain. Moreover, previous studies in *P. aeruginosa* have indeed demonstrated that complementation of mutations in the pyruvate dehydrogenase pathway can be achieved by supplementing acetate in the culture medium (Jeyaseelan and Guest, 1980 doi: 10.1099/00221287-120-2-385, Rae et al., 1997 doi: 10.1128/jb.179.11.3561-3571.1997 and Decheux et al., 2002 doi: 10.1128/IAI.70.7.3973-3977.2002). For this reason, we have metabolically complemented the mutant strains and performed growth analysis and proteomics in presence and absence of acetate (lines 287-293).

Minor concerns:

Line 81: change to "A few examples..."

Changed.

Line 89: change to "if such a mechanism"

Changed.

Line 90: Is undisclosed the right word?

Changed to unexplored.

Lines 134-135 It would also be important to know how many SNP differences were found between each pair of genomes.

We have included this information in Table S1.

Line 180: change to "strain."

Changed.

Line 235-236: What does "directed towards mean"

Changed to 'related to'.

Line 272: The mutations are listed but not the haplotypes. Did each mutation occur in it's own allele or were some found together. Also, define "silent"

We did not find haplotypes for the *aceE* and *aceF* genes. Each mutation occurred independently in each clone type and none of them were found together. We have changed 'silent' to 'synonymous' to avoid confusion.

Lines 409-411. The contention that aceE and aceF mutants occur prior to T3SS mutations is not well supported by the data. This could be addressed using a phylogenetic approach and other existing whole genome sequences.

We agree with the reviewer that our data are not conclusive. Therefore, as also suggested by reviewer 1, we have modified this statement and indicated the need for further investigation. See lines 407-411.

Fig 5g is unclear. Why are the + and o at slightly different heights?

Data are jittered to avoid overlap between symbols and therefore exclusively for visualization purposes.

Reviewer #3:

The evolution of antibiotic resistance dominates the research landscape involving microbial pathogens, but the mechanisms of microbial persistence are similarly important. In this study, the authors examine mechanisms of persistence, with a focus on metabolic rewiring/specialisation. They demonstrate that metabolic rewiring widely occurs during adaptation to the host, and showcase an example that re-wiring metabolism related to pyruvate can enhance the persister phenotype in P. aeruginosa. To achieve this, the authors use a myriad of advanced techniques including metabolomics, proteomics, and confocal microscopy.

Due to the small sample size used in the study (n = 8 for most groupings e.g. 'early vs late' and 'early/late vs PA01') many of the metabolomics and proteomics highlight correlations and trends, but are restricted to broad associations. However the results relating to Figs 5 and 6 - where the authors compare genomic sequence data for 34 patients, make targeted genomic alterations, and assess phenotypes in an environmental context that emulates the human lung - are focussed and highly interesting. In these results the authors show how mutations affecting pyruvate metabolism can directly change phenotypes associated with persistence, showcasing that metabolic rewiring mutations may not only occur to alleviate trade-offs from other mutations.

Overall I feel that the manuscript is timely and presents some exciting results. I have only a few comments and questions regarding the manuscript. I do not believe any of these comments are essential to complete prior to publication, but addressing these may help improve the clarity and readability:

We thank the reviewer for his positive and constructive comments on our manuscript.

1. Lines 90-93: "previous metabolic characterisations of clinical strains were carried out on only a limited number of isolates and/or on bacterial cultures on one specific growth phase". This statement requires citations.

Added.

2. Lines 153-155: "These metabotypes separate the late isolate from each respective early isolate and from the rest of the non-adapted metabotypes". I think using the word 'non-adapted' here is misleading. We do not know if they have not adapted, only that they haven't adapted with regards to the screened phenotypes. Please provide a statement clarifying this next to the 'non-adapted' description.

We agree with the reviewer, so we have modified the sentence to specifically point out that 'non-adapted' refers to metabolic adaptation. See lines 164-166.

3. Lines 178-179: "To test hypothesis that the observed metabolic specialisation is partially rooted in changes in expression of proteins involved in cellular metabolism". Why would the hypothesis be that metabolism changes are only partially rooted in changes to protein expression? This statement seems to be written so that the hypothesis directly matches the results observed, rather than an

organic hypothesis made at the beginning of the experiment. I would suggest either removing the word 'partially' or adding an explanation as to why we would expect proteins relating to metabolism to only partly govern metabolic changes.

We have removed the word partially. See lines 184-186.

4. Lines 179-180: Typo - remove the plural from "we analysed the proteome of each clinical strains".

Changed.

5. Line 284: "Leads to a Phe->Ser amino acid change". Which amino acid position?

Added.

6. Lines 295-299: The authors describe here the lack of association between pathogenic phenotypes and the ace mutations, but they later describe how ace mutants exhibit different persister phenotypes. I was expecting an explanation for these discrepancies to feature more prominently in the discussion.

The lack of association between pathogenic phenotypes and *aceE* and *aceF* mutations refers to the laboratory conditions where most of the phenotypes are usually tested. We have included an explanation of such lack of association in the results (line 295) and discussion (lines 396-398 and lines 470-472).

7. Fig 1: I would suggest swapping the order of Figs 1A and 1B. 1B contextualises 1A by showing the negative correlation between colonisation time in the lung and growth rate, which nicely outlines why we see a difference between the early and late growth rates shown in 1A. I don't see what it adds to the results by going after 1A, however.

We agree with the reviewer that fig 1b contextualises 1a. However, we want to first focus on the general characteristics of the strain collection used in our study and then underline the specific difference between the growth rate of early vs late strains.

8. Fig 2C: Why are there missing datapoints between some early and late conditions?

Some data points and connecting curves are missing because, either the specific metabolites were not assimilated, or the OD_{50} was not possible to calculate since the assimilation window was longer than the time we tested. We have included an explanation in the figure legend. See lines 780-782.

9. Fig 2D: Why are confidence intervals only shown for pyruvate late and formate late? If the confidence intervals fall within the thick lines in the other conditions - or if the other lines have no replicates - please describe this in the figure legend after confidence intervals are discussed.

We realized that the confidence intervals for acetate got missing in the figure. We have now included them.

10. Fig 3E: I would advocate moving this panel to the supplementary information, as I did not find

this association particularly convincing. 740/2061 proteins exhibited differential expression (line 185), of which 235 belonged to those related to metabolism (line 196). When so many proteins exhibit differential expression, we should not be surprised that there are a handful of differences affecting this pathway between clinical strains and a laboratory strain. Similarly, we should also not be surprised to see differences between these strains and those that have spent several more years adapting in a host. How do we know that these changes are all adaptive, could some not be compensatory mutations or products of genetic drift? It may be that the pathways have been altered to "optimise" metabolism in the lung, but as currently presented I think the authors draw too many conclusions from this panel.

As suggested by the reviewer, we have moved figure 3e to supplementary figure S2. We have shortened the corresponding text and introduced a detailed presentation of the proteomic changes at the pathway level in supplementary text 1. Furthermore, we have indicated that further analyses are required to confirm whether changes at pathway level leading to metabolic specialisation are convergent.
